# Infants are sensitive to cultural differences in emotions at 11 months

**DOI:** 10.1371/journal.pone.0257655

**Published:** 2021-09-30

**Authors:** Liquan Liu, Mieke du Toit, Gabrielle Weidemann

**Affiliations:** 1 School of Psychology, Western Sydney University, Sydney, Australia; 2 MARCS Institute for Brain and Behaviour, Western Sydney University, Sydney, Australia; 3 Center for Multilingualism in Society Across the Lifespan, University of Oslo, Oslo, Norway; Bournemouth University, UNITED KINGDOM

## Abstract

A myriad of emotion perception studies has shown infants’ ability to discriminate different emotional categories, yet there has been little investigation of infants’ perception of cultural differences in emotions. Hence little is known about the extent to which culture-specific emotion information is recognised in the beginning of life. Caucasian Australian infants of 10–12 months participated in a visual-paired comparison task where their preferential looking patterns to three types of infant-directed emotions (anger, happiness, surprise) from two different cultures (Australian, Japanese) were examined. Differences in racial appearances were controlled. Infants exhibited preferential looking to Japanese over Caucasian Australian mothers’ angry and surprised expressions, whereas no difference was observed in trials involving East-Asian Australian mothers. In addition, infants preferred Caucasian Australian mothers’ happy expressions. These findings suggest that 11-month-olds are sensitive to cultural differences in spontaneous infant-directed emotional expressions when they are combined with a difference in racial appearance.

## Introduction

Understanding emotion signalling with facial expressions is a critical ability as it plays a crucial role in successful human social communication, interpersonal relationships and even survival [1–6]. Our ability to detect and differentiate facial expressions of emotion has evolutionary benefits in knowing when and how to best respond to displays of aggression or danger [1, 7]. This is especially relevant for infants who have limited resources to derive mental states and expectations of others in their ambient environment. While many have investigated the ontogeny of emotion recognition [8–10], evidence on the extent to which infants perceive the intrinsic cultural elements in emotions remains scarce. The current study examines first-year infants’ sensitivity to racial and cultural information embedded in infant-directed facial expressions.

Debates surrounds the extent to which in infant emotion perception is innate versus learned. On the one hand, universality hypotheses predict that infants should innately recognise the basic universal categories linked to facial expressions [11]. Basic human emotions such as happiness, sadness, fear, anger, surprise, and disgust are hypothesized to have universally recognised facial expressions, innate neural substrates, and unique feeling states [12]. On the other hand, theories of constructed emotions suggest that recognition of (positive vs. negative) valence, the intrinsic attractiveness or aversiveness of emotional expressions [13], and (high vs. low) arousal may be innate [14], but that emotion categories develop slowly and are influenced by experience, culture, and language [15].

There are numerous studies on emotional perception and development in the beginning of life, and findings appear to support both sides of the debate. Newborns and very young infants exhibit an initial preference for happy faces [16–18], but are sensitive to perceptual cues for negative emotions [19, 20], but do not discriminate between fearful and neutral faces [16]. Although newborn infants show no visual preference between happiness and disgust, they are able to discriminate videos of the two emotions if they are habituated to one of the two facial expressions [21]. In 3-month-olds the autonomic nervous system exhibits higher arousal for angry than for happy faces [22], and infants of 5 months are able to discriminate happy and angry facial expressions when matched vocalisations are presented [23]. They also exhibit some degree of neural discrimination between pictures of neutral and fearful faces at this age, but such discrimination appears to be restricted to those who receive relatively high-quality parent-child interactions [24]. White and colleagues [25] report that 5-month-olds exhibit some degree of sensitivity to differentiate between some negative valence emotions such as sadness and disgust, and between positive valence emotions of happiness and surprise, although the same study reports no difference between anger and disgust at 5 or 9 months. The authors argue that sensitivity to emotion categories within the same valence might be due to lower-level perceptual differences in the emotional faces rather than higher-level emotional knowledge of subjective states, which are likely shaped by culture and the development of language. This interpretation is in accordance with a series of studies by Hoehl and colleagues [26–29] reporting infants’ distinct neural responses between angry and fearful faces, which are modulated by social contexts (e.g., eye gaze on referents).

At around 5–7 months, infants shift to a negativity bias [30] and attend more to expressions of fear as compared to [31–34] and when contrasted with happiness [35–37]. However, they prefer objects associated with happy faces over those associated with angry faces [38]. Distinct neural patterns in between happiness, disgust and neutral facial expressions are present among infants of 3.5 and 7 months [39], as well as anger illustrated among 7-month-olds [40]. In an event-related potential (ERP) study, larger negativity to happy than to angry faces is observed in infants at 7 months, and the pattern is reversed at 12 months, indicating successful emotion discrimination and changes in preference over time [41]. Using a preferential looking paradigm where paired pictures of facial expressions are presented side-by-side, 7-9-month-olds look longer at fearful when paired with angry, happy or neutral expressions, while no significant difference is found in between the other pairs [42]. The preferential difference within the negative valence reported in these studies may be a result of the acquisition in the first half of life in addition to the social modulation. Infants of 8–14 months detect threatening stimuli (snakes, angry faces) more quickly than non-threatening ones (flowers, happy faces), but show no preference between happy and fearful facial expressions. This is explained by the difference of directness of threat associated with angry (direct) and fearful (indirect) emotions [43]. Studies using electroencephalogram methods have reported a frontal alpha asymmetry between positive (left activation) and negative (right activation) emotions (for a review, see [44]). By the end of the first year after birth, infants can discriminate angry from neutral [45], and other negative facial expressions such as fear [46, but see 47] and pain [48].

Irrespective of the debate on the inner state of emotion categories, there is no dispute that emotional expressions are modified by socio-cultural learning, such as rules and norms shaped via interpersonal experience, family context and social environments [49–57]. The neurocultural theory of emotional expression [1] states that facial prototypes of emotions are stored innately and combined with culturally learned display rules which dictate the modification of expression and the perception of others’ emotion. Studies have reported that emotions expressed by members of the same national, ethnic, and regional cultural “in-group” are better recognized than those expressed by “out-group” [3, 58, 59]. The cues people adopt to communicate emotions vary substantially across cultures from distinct facial movements [49] to the mental state or situated behavioural inferences [60].

The cross-cultural discrepancies in the specific facial cues and temporal dynamics of those cues are most apparent between Western Caucasian and East Asian cultures, with discernable differences in facial expressions of emotion appearing in 3-year-old East Asian participants adopted into Western Caucasian families [61, 62]. Having said that, a recent study reported longer gaze at fearful and shorter gaze at angry faces for both German and ≠Akhoe Hai||om children and adolescents aged between 7 and 19 years, pointing to the commonality in emotion perception across cultures [63].

Until now only a handful of studies have examined how culture influences infants’ recognition and knowledge of emotion categories. Geangu and colleagues [64] explored visual scanning patterns of 7-month-old Western Caucasian and East-Asian infants towards photos showing culture-specific facial expressions using a visual discrimination paradigm. Although mothers’ race and (happy or fearful) facial expressions do not alter infants’ looking patterns, there are visual biases to the mouth area for Western Caucasians as compared to East-Asians who focus longer on the eye region. This finding matches the evidence of differences in the specific facial signals and temporal dynamics of different emotion expressions between Western Caucasian and East-Asian cultures [62]. Although the study suggests that infants growing up in different cultures may find different areas of the face more informative, whether infants can discriminate between certain emotions between different cultures remains unknown.

In the investigation of the early development of emotional processing, previous research has focused predominantly on infants’ perception of static or reduced facial expressions. While such design may facilitate the examination of certain specific parameters or questions, the detailed type and amount of information embedded in the emotions, such as intensity, are likely to be restricted and incomplete [65]. Naturally moving faces can invite more attention and more elaborative processing [66, 67], supplying emotion perception studies with more ecologically valid stimulus materials [68, 69]. Nevertheless, how dynamic information modulates infants’ responses has rarely been investigated [48, 70, 71], and this is even rarer when taken natural mother-infant expressions into consideration [72], given that infant-directed facial expressions differ from adult-directed expressions [73].

As the acquisition of one’s culture is arguably a gestalt process following various cues in the ambient environment, the present study is the first to adopt spontaneous, infant-directed facial expressions. It examines whether infants are sensitive to cultural differences in emotions through their preferential looking patterns at paired videos of emotions expressed by mothers from different cultures. There is considerable evidence suggesting that similar to the way that human speech directed at infants is modified, a phenomenon known as “baby talk”, parents modify their faces in special ways when interacting with infants [73]. In other words, the way that facial expressions of emotion are displayed to infants (infant-directed) can differ from the way in which these emotions are displayed to adults (adult-directed). Chong, Werker, Russel and Caroll [74] identify three distinct infant-directed facial expressions, comfort/caring, surprise/interest, and happiness, among Chinese- and English-speaking mothers living in Canada, and suggest that these infant-directed facial expressions may be universal across cultures. However, an assessment of whether infants differentiate between and/or prefer infant-directed facial expressions based on cultural backgrounds would serve to determine whether these expressions are in fact universal.

This study used facial expressions from infants’ own (Australian) and an East-Asian (Japanese) cultures, as there is substantial evidence for cultural differences in emotion display rules [59, 75–78, but see 79]. However, when comparing Australian with Japanese facial expressions, an evident confound in racial appearances [80], and there is considerable evidence showing that infants differ in their preference and recognition for own- and other-race faces (for a review, see [81]). In order to control for this, we employed own-culture models who matched infants’ race (Caucasian Australian) as well as mothers who did not (East-Asian Australian). Consequently, the comparison between Caucasian and East-Asian Australian mothers is a comparison of race, the comparison between East-Asian Australian and Japanese mothers is a comparison of culture in the absence of own race, and the comparison between Caucasian Australian and Japanese mothers is a comparison of differences in both race and culture. This design allows us to tease out the effects of both differences in racial appearance as well as differences in cultural expression of the different infant-directed emotions that the infants are sensitive to. To test whether emotions identified by Chong et al. [74] are universal, we chose to examine cultural differences in happy, surprised and angry expressions, as happy and surprised were identified as being similar across cultures but no universal angry infant-direct facial expression was observed. If there are universal infant-directed facial expressions of happiness and surprise, infants may not detect cultural differences in the display of these emotions but they may prove sensitive to cultural differences in the facial expression of anger. On the other hand, Jack and colleagues [62] have shown that adult-directed happy, surprised and angry facial expressions all differed in muscle movements around the eyes between East-Asian and Western Caucasian cultural expressions, and happy expressions also differed in muscle movements in the cheeks. If these expressions also differ between cultures in their infant-directed forms, infants may detect cultural differences in the display of all three emotions.

We hypothesized that by year one, Caucasian Australian infants would be sensitive to not only racial information but also culture-specific displays. Specifically, infants would show differences in looking times for expressions from their own culture compared to those of another culture and such difference would be exaggerated by differences in race. In addition, we predicted that infants would be more likely to show sensitivity to cultural differences in angry than happy and surprised expressions, as the latter two may be more culturally universal, particularly when directed towards infants.

## Method

### Participants

The final sample consisted of 23 participants (*M*_age_ = 11.73m, *SD*_age_ = 0.47m, *N*_female_ = 14) recruited from the Western Sydney University Babylab Register. This number is in line with the minimally required total sample size (*N* = 22) from G-power analysis [82] with three types of mothers * three types of emotion of repeated measures analysis of variance and moderate effect size (*f* = 0.25) on within-subject infant looking time measures [83]. All infants are full-term, typically developing, and growing up in Caucasian families in Australia with limited exposure to East-Asian faces and cultures. Three additional participants were tested but not included in the final sample due to: age too young for the group (1) and technical errors during the experiment (2). Participants were recruited and tested under the research project (H11387) that was approved by Western Sydney University Human Research Ethics Committee, with written consent forms obtained from parents or guardians before the experiment.

### Stimuli

Mothers from two cultural backgrounds (Australian vs. Japanese) and racial (Caucasian vs. East-Asian) groups were invited to the Babylab. All mothers were born, raised and lived in the corresponding cultures, and Japanese mothers relocated from Japan to Australia recently and were recruited from a local Japanese mothers’ get-together group. These variables formed three types of cultural/racial categories: Caucasian Australian, East-Asian Australian and Japanese. Luminance was kept constant in the recordings across participants and emotions. All mothers wore the same shirt and removed all accessories (e.g., jewels). A chair was placed directly against a white wall at its back and one meter away from the camera at its front. Mothers sat on the chair facing their infants, who were seated on a baby chair in between the chair and the camera. The shot was framed so that the baby’s head did not appear in the frame. Mothers were asked to recall real-life situations leading to three types of spontaneous infant-directed emotions (anger, happiness, and surprise) to their children and their facial expressions were recorded. When asked to illustrate surprise to infants, mothers unanimously expressed pleasant/interested surprise emotions shown in Chong et al. [74] rather than fearful surprise. Although mothers exhibit some degree of motions in their facial expressions, previous studies suggest that infants’ enhanced attention to facial expressions was not due to low-level features like movement [31, 32, 84]. Crucially, previous studies focus on comparisons between emotions (e.g., positive vs. negative valence), and we compared the *same* emotion across cultures. Compared to Japanese mothers, Australian mothers illustrated slightly more movements in their facial expressions. As our goal was to investigate infants’ perception of spontaneous, infant-directed emotions, potential distracting facial features in the external portion of the face (e.g., hair, [85]) were kept to maintain stimulus naturalness. Previous studies have reported that infants’ attention is unlikely to be affected by these features when emotions are in play [31, 32].

To control for stimuli-induced effects, two mothers were recorded in each type of emotional and cultural/racial categories, making up 18 videos. Each emotional clip was formatted to 3-second recordings with mothers’ faces in the centre of the screen, from neutral facial expressions at the first frame immediately shifting towards the target emotion which continues throughout the clip. With respect to stimuli physical features, mean motion across time over the video was examined [84]. In general, Japanese mothers exhibited less motion content in happiness and anger than, but comparable motion content in surprise as Australian mothers. (See S1 File for details). These videos were further rated by 19 Caucasian Australians (Mean_age_ = 26.3 years, SD_age_ = 8.91 years) and 10 Japanese (Mean_age_ = 25.5 years, SD_age_ = 7.47 years) adults, with significant above-chance goodness of fit ratings (*p* < .001) for the corresponding emotional categories. In the attractiveness ratings, on the other hand, a hierarchical preference for happiness > surprise > anger was observed (*p* < .001) across subjects. The racial and cultural backgrounds of neutral-emotion pictures of the Japanese and East-Asian Australian mothers were also judged by another 10 Caucasian Australian adults (*N*_female_ = 4, Mean_age_ = 27.9 years, SD_age_ = 4.67 years) in a forced choice task. Adults had no problem judging that mothers’ race was East-Asian rather than Caucasian (Mean_accuracy_ = 97.5%, SD_accuracy_ = 7.9%), but could not tell whether these mothers were from Australian or Japanese backgrounds (Mean_accuracy_ = 50%, SD_accuracy_ = 11.8%). Each 3-second clip was self-repeated for three times, resulting in 9-second videos used as the final stimuli of the current study.

### Procedure

A 3-screen preferential looking paradigm was adopted and stimuli were presented via 40 * 30 cm flat-screen LG monitors. Infants sat on caretakers’ laps on a chair approximately 60 centimetres from the middle screen. Each participant was presented with 18 trials. The trials started when infants maintained attention for 2 secs on the attention-getter (graphic clips with sounds) appearing on the middle screen. During each trial, two videos from the same emotional category but different cultural/racial conditions were presented on the left and right screens simultaneously. There were 6 trials with Caucasian Australian and East-Asian Australian mothers, 6 trials with Caucasian Australian and Japanese mother and 6 trials with East-Asian Australian and Japanese mothers, two trials of each of emotion angry, happy and surprised for each cultural/racial comparison, such that each cultural/racial category appeared once on the left and the right for each emotion. Two different pseudo-random trial orders were used across participants with the restriction that no more than two trials with the same emotion and/or the same cultural/racial comparison could appear consecutively. Infants’ looking preferences and time were coded by an experimenter in an adjacent room. Participating families received travel reimbursement, certificates of participation, and age-appropriate toys after the experiment.

## Results

To examine whether the type of comparison face influenced looking times, the percentage of looking times at the three different face types (Caucasian Australian, East-Asian Australian and Japanese) as a function of the overall time spent looking at the two videos was calculated. An independent researcher rated 10% of the recordings with an inter-rater agreement of 95%. Results were submitted to linear mixed-effects modelling analyses using the lme4 [86] package’s lmer function in R [87]. Three sets of analyses were conducted corresponding to the proportion of looking time towards each of the three trial types (Caucasian Australian, East-Asian Australian, and Japanese). Within each analysis, Pair (2-level, in which the examined *Trial type* occurred) and *Emotion* (3-level, angry, happy, surprised) were included as the fixed factors while *Participant* was set as the random factor. Factors were examined in a cumulative manner, building from the simplest model (1) and compared with a more complex model. The most complex model was listed (2). Post-hoc effects were examined under pairwise comparisons using *emmeans* (e.g., (3), corresponding to (2)). The commands were:

lmer(Prop.LT~(1|Participant),data=data) (1)

lmer(Prop.LT~Pair*Emotion+(1|Participant),data=data) (2)

emmeans(model,list(pairwise~Pair|Emotion)) (3)

### Proportion of looking towards Caucasian Australian faces

**Fig 1:**
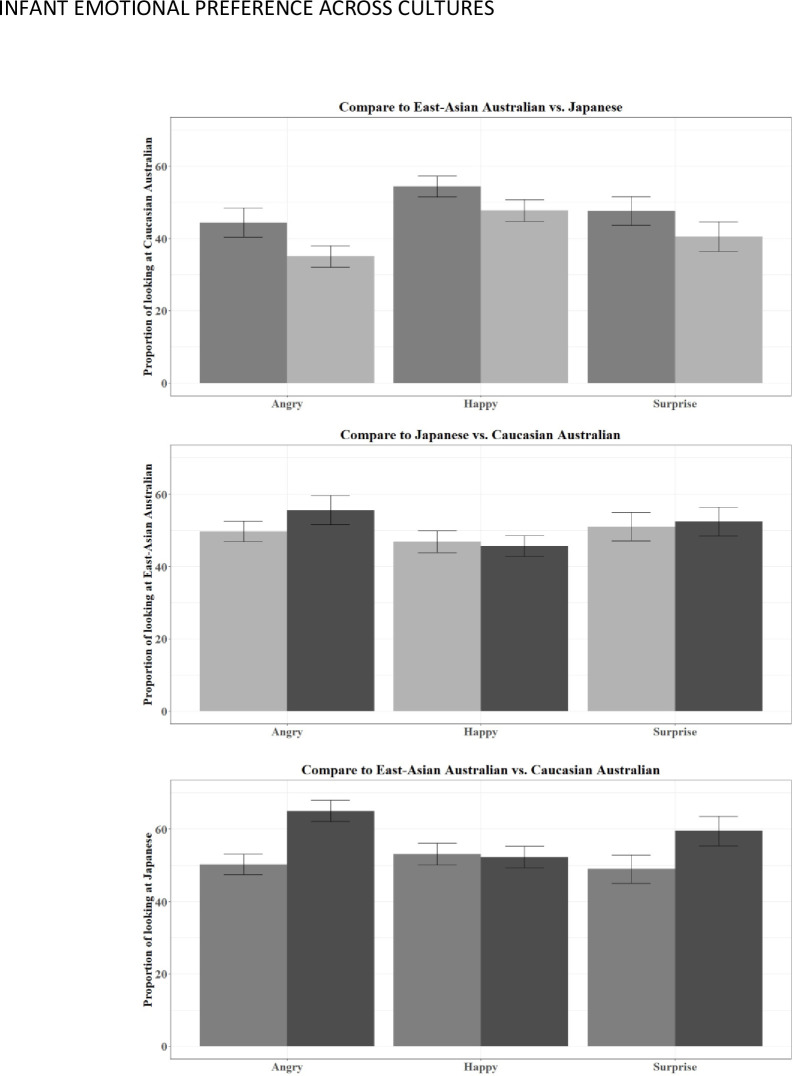
Proportion of looking towards Caucasian Australian (top panel), East-Asian Australian (middle panel) and Japanese (lower panel) faces when paired with the other two cultural/racial face types (Japanese in light grey, East-Asian Australians in medium grey, and Caucasian Australian in dark grey) in angry, happy and surprised facial expressions. Error bar: ± lSE.

The proportion of looking time at Caucasian Australian mothers was analysed in linear mixed-model analysis as a function of the Trial type (compared with East Asian Australian and Japanese) and the type of Emotion being expressed (Angry, Happy, Surprised). Trial type (estimate = 10.19, SE = 4.85, df = 245, t = 2.10, *p* = .003) and emotion (estimate = 9.77, SE = 4.91, df = 245, t = 1.99, *p* = .004) but not their interaction (estimate = 2.91, SE = 6.86, df = 244.62, t = 0.42, *p* = .879) were significant predictors of the model. For Trial type, post hoc pairwise comparisons revealed significant shorter proportion of looking times towards Caucasian Australian mothers when paired with Japanese mothers than when paired with East-Asian-Australian mothers (estimate = 8.49, SE = 2.84, df = 233, t = 2.98, *p* = .003). With respect to Emotion, proportion of looking time towards Caucasian Australian mothers was significantly lower in Angry than Happy trials (estimate = 10.66, SE = 3.50, df = 234, t = 3.048, *p* = .007), and not significant in Angry vs. Surprised (estimate = 4.41, SE = 3.48, df = 233, t = 1.27, *p* = .415) or Happy vs. Surprised (estimate = 6.26, SE = 3.48, df = 233, t = 1.280, *p* = .172) comparisons (Fig 1).

### Proportion of looking towards East-Asian Australian faces

The proportion of looking time at East-Asian Australian mothers was analysed in linear mixed-model analysis as a function of the trial type (compared with Caucasian Australian and Japanese) and the type of emotion being expressed (Angry, Happy, Surprised). None of Trial type (estimate = 4.91, SE = 4.94, df = 265, t = 0.99, *p* = .859), Emotion (estimate = 2.82, SE = 74.89, df = 265, t = 0.58, *p* = .213), or their interaction (estimate = 6.73, SE = 6.97, df = 265, t = 0.97, *p* = .746) reached statistical significance (Fig 1).

### Proportion of looking towards Japanese faces

The proportion of looking time at Japanese mothers was analysed in linear mixed-model analysis as a function of the trial type (compared with Caucasian Australian and East-Asian Australian) and the type of emotion being expressed (Angry, Happy, Surprised). Trial type (estimate = 14.68, SE = 4.72, df = 247, t = 3.11, *p* = .003) and its interaction with Emotion (estimate = 15.51, SE = 6.66, df = 246.65, t = 2.33, *p* = .054) but not Emotion (estimate = 2.84, SE = 4.72, df = 247, t = 0.60, *p* = .313) were significant predictors of the model. Post hoc pairwise comparisons revealed significant longer proportion of looking times towards Japanese mothers when paired with Caucasian-Australian mothers than when paired with East-Asian-Australian mothers, in Angry (estimate = 14.68, SE = 4.72, df = 247, t = 3.11, *p* = .002) and Surprised (estimate = 10.50, SE = 4.69, df = 247, t = 2.236, *p* = .026) but not Happy (estimate = 0.83, SE = 4.69, df = 247, t = 0.18, *p* = .859) emotions (Fig 1).

To sum up, in trials where Caucasian Australian mothers were present, infants revealed a general preference for looking at Japanese mothers and a general preference in happiness. In trials where East-Australian mothers were present, no robust preference was observed across trial types or emotions. In trials where Japanese mothers were present, preferences were observed over Caucasian Australian mothers in anger and surprise but not in happiness.

## Discussion

Human cognitive mechanisms have been argued to be predominantly shaped by culture [88]. The current study examined 11-month-old Caucasian Australian infants’ preferences for spontaneous, infant-directed cross-cultural emotional expressions. Distinct patterns for each emotion emerge from the current findings: First, in trials with Caucasian Australian mothers, infants looked less at the Caucasian mothers when paired with Japanese mothers than when paired with East-Asian Australian mothers, and preferred the happy expressions over the angry expression. No trial type or emotion difference was observed in trials involving East-Asian Australian mothers. Second, in trials with Japanese mothers, there was a preference for looking at the Japanese mothers when paired with Caucasian Australian than with the East-Asian Australian mothers, for the angry and the surprised but not for happy facial expressions of emotion.

Infants demonstrated elevated looking for Japanese over Caucasian Australian mothers’ angry and surprised emotional expressions. The two mothers’ groups differed in both racial and cultural profiles. A comparison of looking times on paired trials differing only by race (Caucasian Australian vs. East-Asian Australian) or only by culture (Japanese vs. East-Asian Australian) showed no looking preference. These results indicate that 11-month-old Caucasian Australian infants were sensitive to the differences in the display of infant-directed emotions between Australian and Japanese cultures, as their looking times at the Caucasian Australian mothers varied depending on the culture of the comparison face while the difference in the race (East-Asian) of the comparison face was held constant. Thus, sensitivity is only observed when cultural differences are combined with differences in racial appearance.

By age one, infants appear to be sensitive to cultural differences in the portrayal of some emotions. Previous studies have reported that adults and children as young as 3 years recognize emotional categories more accurately when portrayed by individuals from their own culture compared with other cultures [59, 89, 90]. The current findings provide empirical evidence to theories claiming emotions are perceived during infancy [28] and suggest that differences in emotional expressions between cultures ultimately influence emotion recognition. That is, infants’ sensitivity to cultural differences in the expression of emotion may be a precursor to differences in recognition of emotional categories across cultures.

The current research also extends on infant race/face perception and preferences. Specifically, there was evidence for a preference based on race, in that there was a difference in looking time at the Japanese mothers depending on the race of the comparison face, while the difference in the cultural background of the comparison face was held constant (Australian). There is considerable evidence to suggest that newborn infants show no preference for faces on the basis of race but that 3-to-12-month-olds show an own-race preference based on greater exposure to own-race faces [91–93]. Some recent studies have shown that 9-month-olds prefer other-race faces after passing through a null-preference period at 6 months [94, 95]. Notably, the preference for other-race face present in our 11-month-old sample was mostly observed when differences in race were combined with differences in culture, but not when the faces were from the same culture.

Consequently, race cannot be the sole cue affecting culture-specific knowledge as no difference was observed in looking time at trials of Caucasian and East-Asian Australian mothers. Instead, the current findings indicate a novelty preference for other culture over infants’ own cultural when combined with a difference in race. Among various perceptual sensory cues, some intrinsic properties, such as the salience of facial expressions, may play a role in preference. Emotions across cultures differ in their intensity, and infants’ sensory space can be elaborated based on physical properties that hold sensory salience [96, 97]. The direction of infant cultural preference shown in the current study is thus of particular interest. Japanese infant-directed expressions of anger and surprise appear to be preferred over Australian infant-directed expressions. In the speech domain, infants tend to prefer cues with strong salience. For example, infant-directed speech, consisting of a higher degree of positive affect and hyper-articulation, are preferred over adult-directed speech from the first month after birth [98, 99]. If sensory salience plays a role, we would expect that infants prefer a stronger degree of emotions, apparent in the Australian compared to Japanese expressions. This is opposite to what we found. If infants’ focus on Japanese anger is due to avoidance of stronger negative emotion, the same explanation cannot account for their focus on (positive) surprise. Similar to race, sensory salience cannot be the only factor that explains the current finding. Australian infants must be sensitive to the different cultural expressions embedded in the emotional output and displayed a novelty preference for Japanese expressions. Further research could explore visual cues infants utilise in order to differentiate emotion expressions between cultures.

Last but not least, there was limited evidence of preference on trials where a happy emotional expression was displayed across races and cultures, except when it is expressed by Caucasian Australian mothers. One explanation could be that there is a more universal infant-directed happy expression as identified by Chong et al. [74]. Though not representable from our data, it has been argued that attention to happiness may be more flexible across different cultures [63]. Another explanation comes from a recent study [100] where participants’ emotion detection ability is examined using a perceptual learning procedure. Results show that training on any of the six basic emotions would improve the detection of happiness, suggesting that these emotions may share some common underlying psychological components. A third explanation would be that happiness is arguably the most frequent emotions infants experience since birth. It has been argued that normal development may require a positive context in which infant largely experience positive emotions and interaction [18]. Compared to results of the other emotions which may reflect a novelty effect, the null results across racial and cultural types and the general trend towards Caucasian Australian mothers reflect infants’ familiarity with happiness and greater exposure to variance in the expression of this emotion in general, as well as its embedded racial-cultural information in specific.

This study was the first to examine infant cross-cultural emotional perception using spontaneous infant-directed facial expressions. We believe this practice can lead to advantages in ecological validity, but acknowledge related limitations in result interpretation. First, to obtain spontaneous, natural emotional expressions, recorded mothers were instructed to interact with infants face-to-face, leading to a 45°-downward eye gaze pattern. The directness of gaze could affect infants’ emotional processing of facial expressions [101], and we predict direct eye gaze would lead to more robust findings of the current patterns. Second, as dynamic videos were used instead of static pictures, it is reasonable to discuss whether differences in movement would promote attention/attraction and affect the current findings [31, 32, 84]. Considered as part of the cultural display, movements were not controlled across cultures in the study. Japanese mothers’ movements were more constrained than Australian mothers, yet their angry/surprise emotions were preferred, opposite to the movement-promotion hypothesis. Third, external facial features were included, different from some studies [70] but comparable with others [42]. Although external features can attract infants’ attention [85], eye-tracking studies suggest that infants focus on eyes and mouth areas in emotional perception [64]. We used multiple templates in the current study to increase variability, and leave these and other possible influential factors open to future research.

## Conclusion

Greater attention to culture has been called for in developmental research [102]. The current study demonstrates 11-month-old infants’ sensitivity to differences in culture-specific emotional expressions when combined with differences in racial appearance, but that this sensitivity is more apparent for anger and surprise (novelty effect) than for happiness (familiarity effect). Findings indicate that infant socio-emotional development is a cumulative environmental learning process beginning early in life. Future studies should investigate the development of cultural preferences by testing infants across ages and cultural backgrounds.

## Supporting information

S1 File (DOCX)
