# Supplementary material for: Infants are sensitive to cultural differences in emotions at 11 months
Source: PLoS One. 2021 Sep 30;16(9):e0257655. doi: 10.1371/journal.pone.0257655 (PMC8483341; doi:10.1371/journal.pone.0257655)
Supplement: S1 File — (DOCX) [file pone.0257655.s001.docx]

**S1 File**

The figures below displayed mean motion content change (quantified as change in luminance per pixel) plotted across time over the emotion for each type of mothers (different lines) and emotion (different plots). The method was used to examine stimuli physical features of video displays of emotion (Grossmann & Jessen, 2017; Heck, Hock, White, Jubran, & Bhatt, 2016; 2017). In figures, AA (blue, solid line) refers to East-Asian Australian mothers, AJ (red, dotted line) refers to Japanese mothers, and CA (green, dashed line) refers to Caucasian Australian mothers.

ANOVA has been conducted across three types of mothers for each emotion. Tests of between-subjects effects showed significant differences in between mother types in anger (F (2, 204) = 64.815, *p* < .001,  *η*_p_^2^ = .389) and happiness (F (2, 204) = 56.231, *p* < .001,  *η*_p_^2^ = .355) but not surprise (F (2, 204) = 2.489, *p* = .086,  *η*_p_^2^ = .024). Post hoc analyses showed that motion contents from Japanese mothers were less than Australian mothers in angry and happy expressions (*p*s < .001).


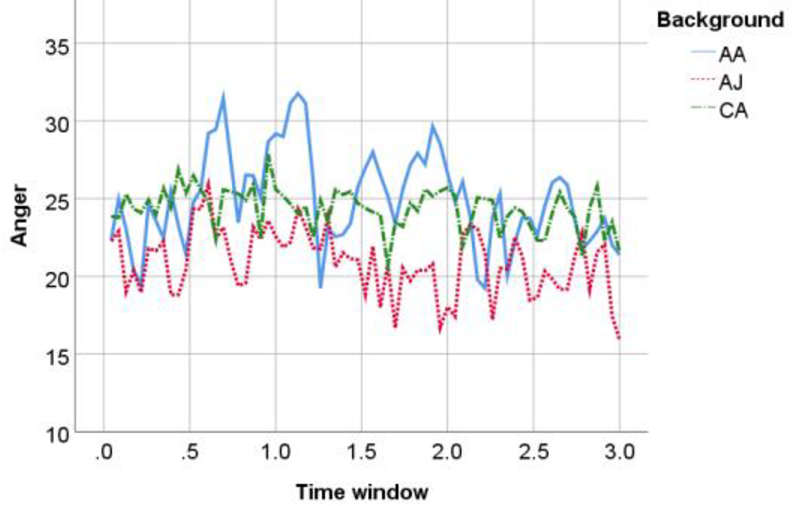


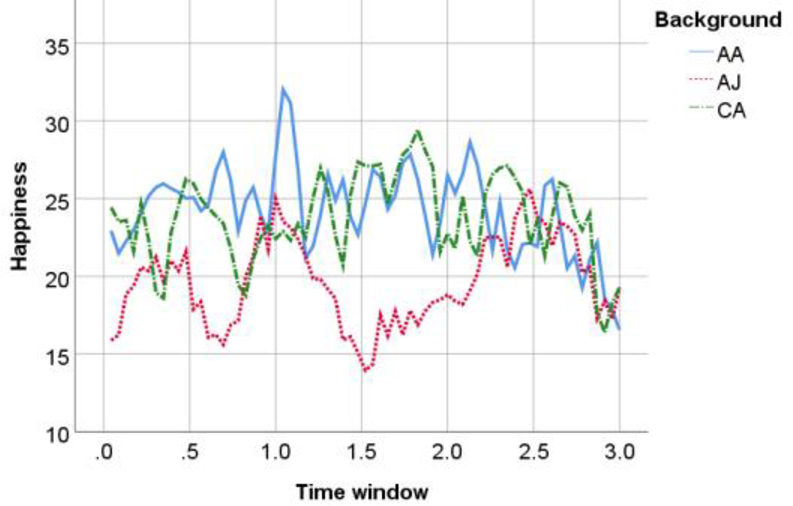


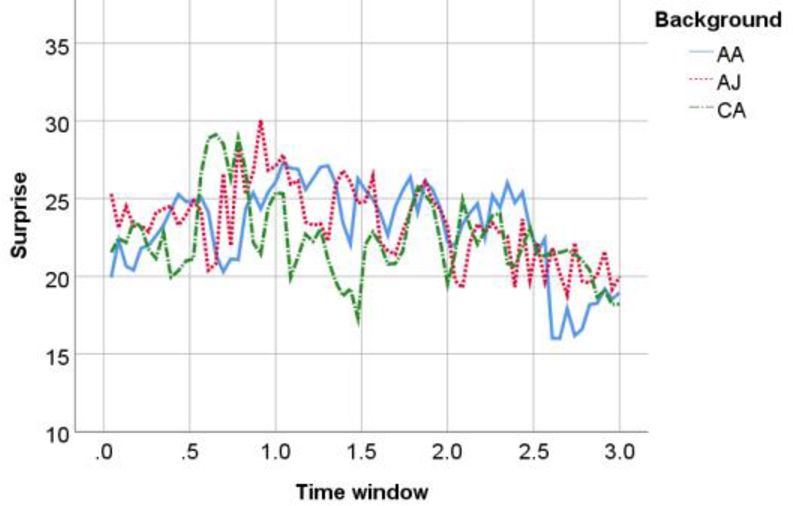


**References**

Grossmann, T., & Jessen, S. (2017). When in infancy does the “fear bias” develop?. *Journal of Experimental Child Psychology*, *153*, 149-154.

Heck, A., Hock, A., White, H., Jubran, R., & Bhatt, R. S. (2016). The development of attention to dynamic facial emotions. *Journal of Experimental Child Psychology*, *147*, 100-110.

Heck, A., Hock, A., White, H., Jubran, R., & Bhatt, R. S. (2017). Further evidence of early development of attention to dynamic facial emotions: Reply to Grossmann and Jessen. *Journal of experimental child psychology*, *153*, 155-162.
